# Etymologia: Prion

**DOI:** 10.3201/eid1806.120271

**Published:** 2012-06

**Authors:** Lawrence B. Schonberger, Robert B. Schonberger

**Affiliations:** Centers for Disease Control and Prevention, Atlanta, Georgia, USA (L.B. Schonberger);; Yale University School of Medicine, New Haven, Connecticut, USA (R.B. Schonberger)

**Keywords:** prion, Etymologia, transmissible spongiform encephalopathies, viruses

**To the Editor:** The January 2012 Etymologia section might confuse readers because it incorrectly reports that “prion” describes a noninfectious agent (*1*). In fact, prion—pronounced *pree'-on*—is a term coined in 1982 by Nobel laureate Stanley Prusiner to describe the novel infectious agent responsible for scrapie, a transmissble neurodegenerative disorder of sheep and goats. He proposed his new term to underscore that the agents are “small ***pro***teinaceous ***in***fectious particles” resistant to procedures that attack nucleic acids (*2*). In his seminal article, he summarized experimental data indicating that the molecular properties of this infectious agent differed from those of other infectious agents, including viruses, viroids, and plasmids; he proposed the word prion to replace other terms then in circulation, such as “unconventional virus” or “unusual slow virus–like agent.”

Although Dr. Prusiner acknowledged that he could not exclude the possibility of a small nucleic acid contained within the interior of the prion particle, now 3 decades later, no nucleic acid in the agent has yet been identified. Increasingly accepted in the scientific community, prions are now considered to be a class of misfolded proteinaceous, infectious agents responsible for several types of human and animal transmissible spongiform encephalopathies. Their evolving defining characteristics classically include at least partial protease resistance, insolubility, and transmissibility. The term, prions, usually refers to the complete transmissible proteinaceous particles in nature or to their classically present, transmissible, protease-resistant oligomer cores, composed of protein fragments with molecular masses of ≈27–30 kDa.

Adding confusion to the terminology, it has become customary for prion researchers to refer to the normal nonpathogenic conformation of prions as “cellular prion proteins” (*3*). When these normal cellular prion precursors convert to pathogenic prion proteins, the transmissible conformations are characterized by β-pleated sheets rather than the normal α-helix structure, and they do not elicit an immune response (*4*).
